# Correction: Age and political leaning predict COVID-19 vaccination status at a large, multi-campus, public university in Pennsylvania: A cross-sectional survey

**DOI:** 10.1371/journal.pone.0322942

**Published:** 2025-04-22

**Authors:** Ryan Murphy, Lauren Pomerantz, Prabhani Kuruppumullage Don, Jun Sung Kim, Bradley A. Long

The following information is missing from the Data Availability statement: Long, Bradley; Murphy, Ryan; Pomerantz, Lauren; Kuruppumullage Don, Prabhani (2023), “Pennsylvania State University Fall 2021 COVID-19 Infodemic Survey”, Mendeley Data, V2, https://doi.org/10.17632/vvnsj7n56t.2

The Funding statement for this paper is incorrect. The correct Funding statement is as follows: The project was funded by an intramural grant from the Pennsylvania State University Libraries in the amount of $2105. Additionally, RM and LP received compensation as research assistants from the Harrell Health Sciences Library at the Penn State College of Medicine.

## References

[1] Murphy R, Pomerantz L, Kuruppumullage Don P, Sung Kim J, Long BA. Age and political leaning predict COVID-19 vaccination status at a large, multi-campus, public university in Pennsylvania: a cross-sectional survey. PLoS One. 2023;18(9): e0291974. doi: 10.1371/journal.pone.0291974 37729180 PMC10511139

