# Supplementary material for: Age and political leaning predict COVID-19 vaccination status at a large, multi-campus, public university in Pennsylvania: A cross-sectional survey
Source: PLoS One. 2023 Sep 20;18(9):e0291974. doi: 10.1371/journal.pone.0291974 (PMC10511139; doi:10.1371/journal.pone.0291974)
Supplement: S1 File — (PDF) [file pone.0291974.s001.pdf]

Logistic Analysis

0.0.1 Front matter

Here we load tha data and the libraries we use for the analysis.

```
# dimensions of the data set read
dim(origData)
```

```
[1] 4231  107
```

0.0.2 Data cleaning for logistic regression analysis

Here, the independent variables of interest are *age*, *gender* and *political leaning*, and the reponse variable is *vaccine status for COVID-19*.

We will first look at the predictors, starting from *gender*.

```
# Gender
origData <- apply_labels(origData, gender_id=c("Man" = 1,
                              "Woman" = 2, "Other" = 3, "Prefer not to answer" = 4))
table(origData$gender_id, useNA = "always")
```

```
                 Man                Woman                Other 
                1584                 2308                   58 
Prefer not to answer                 <NA> 
                  85                  196 
```

We combine the levels *other* and *prefer not to answer* to create a new category.

```r
# Gender - regrouping
origData$gender_id[origData$gender_id == 4] <- 3
origData <- apply_labels(origData, gender_id=c("Man" = 1,
                                 "Woman" = 2, "Other/Prefer not to answer" = 3))
table(origData$gender_id, useNA = "always")
```

```
                      Man                       Woman
                     1584                        2308
Other/Prefer not to answer                       <NA>
                      143                         196
```

The next independent variable of interest is *age*.

```r
# Age
origData <- apply_labels(origData, age_range=c("18-20" = 1,
                                 "21-25" = 2, "26-35" = 3, "36-45" = 4,
                                 "46-55" = 5, "56-65" = 6, "Older than 65" = 7,
                                 "Prefer not to answer" = 8))
table(origData$age_range, useNA = "always")
```

```
             18-20                21-25                26-35
               695                  728                  851
             36-45                46-55                56-65
               626                  575                  458
     Older than 65 Prefer not to answer                 <NA>
                79                   31                  188
```

```r
# Age - regrouping
origData$age_range[origData$age_range == 7] <- 6
origData$age_range[origData$age_range == 8] <- 7
origData <- apply_labels(origData, age_range=c("18-20" = 1,
                                 "21-25" = 2, "26-35" = 3, "36-45" = 4,
                                 "46-55" = 5, "older than 55" = 6, "Prefer not to answer" = 7)
table(origData$age_range, useNA = "always")
```

```
             18-20                 21-25                 26-35 
               695                   728                   851 
             36-45                 46-55         older than 55 
               626                   575                   537 
Prefer not to answer                  <NA> 
                31                   188 
```

The last predictor of interest is *political leaning*.

```
# Political Leaning
origData <- apply_labels(origData, political_leaning=c("Far left" = 1,
                             "Liberal" = 2, "Middle-of-the-road" = 3, "Conservative" = 4,
                             "Far right" = 5, "Prefer not to answer" = 6))
table(origData$political_leaning, useNA = "always")
```

```
            Far left               Liberal   Middle-of-the-road 
               290                  1271                  1141 
        Conservative             Far right Prefer not to answer 
               766                    68                   325 
              <NA> 
               370 
```

The response variable of interest in our study is *vaccine status for COVID-19*.

```
# Vaccination status
origData <- apply_labels(origData, covid_vaccination_status=c("Yes" = 1,
                             "No" = 2, "Undecided" = 3, "Prefer not to answer" = 4))
table(origData$covid_vaccination_status, useNA = "always")
```

```
                 Yes                    No             Undecided 
                2995                   520                   126 
Prefer not to answer                  <NA> 
                  63                   527 
```

We decided to work with complete cases and also focus on the cases with responses who answered yes/no to either having received or planning to receive COVID-19 vaccine. We also removed the responses who identified other for gender or preferred not to answer, and who

preferred not to disclose the political leaning. We also removed the cases that preferred not to report their age group.

```
#removing NAs to get complete cases
origData <- origData[!((is.na(origData$gender_id))|(is.na(origData$age_range))|
                          (is.na(origData$political_leaning))|
                          (is.na(origData$covid_vaccination_status))),]
# removing cases undecided and prefer not answer on the vaccine status
origData <- origData[(origData$covid_vaccination_status==1)|(origData$covid_vaccination_st
# removing cases marked other and prefer not answer on gender
origData <- origData[(origData$gender_id==1)|(origData$gender_id==2),]
# removing cases marked prefer not answer on political leaning
origData <- origData[(origData$political_leaning!=6),]
# removing cases marked prefer not answer on age
origData <- origData[(origData$age_range!=7),]


#table(origData$gender_id)
table(origData$covid_vaccination_status)
```

```
 Yes   No 
2713  428 
```

The data cleaning process leaves us 3465 complete cases.

0.0.3 Chi-square test results for Associations

```
#vaccine status vs political leaning
chisq.test(origData$covid_vaccination_status, origData$political_leaning)
```

```

	Pearson's Chi-squared test

data:  origData$covid_vaccination_status and origData$political_leaning
X-squared = 514.18, df = 4, p-value < 2.2e-16
```

```r
#vaccine status vs age
chisq.test(origData$covid_vaccination_status, origData$age_range)
```

```
	Pearson's Chi-squared test

data:  origData$covid_vaccination_status and origData$age_range
X-squared = 70.097, df = 5, p-value = 9.783e-14
```

```r
#vaccine status vs gender
chisq.test(origData$covid_vaccination_status, origData$gender_id)
```

```
	Pearson's Chi-squared test with Yates' continuity correction

data:  origData$covid_vaccination_status and origData$gender_id
X-squared = 3.1934, df = 1, p-value = 0.07394
```

0.0.4 Logistic regression analysis

```r
# relabeling vaccine status to model "yes" vs "no"
origData$covid_vaccination_status[(origData$covid_vaccination_status==2)] <- 0
origData <- apply_labels(origData, covid_vaccination_status=c("Yes" = 1,
                             "No" = 0))
table(origData$covid_vaccination_status, useNA = "always")
```

```
  No  Yes <NA> 
 428 2713    0 
```

```r
# ensuring the features are recognized as factors
modelData <- lapply(origData[,c("covid_vaccination_status", "age_range",
                              "political_leaning", "gender_id")],
                    factor)
str(modelData)
```

```
List of 4
 $ covid_vaccination_status: Factor w/ 2 levels "No","Yes": 2 2 2 2 2 2 2 2 1 2 ...
 $ age_range               : Factor w/ 6 levels "18-20","21-25",..: 5 4 3 4 5 6 6 6 1 1 ...
 $ political_leaning       : Factor w/ 5 levels "Far left","Liberal",..: 2 3 2 2 3 3 2 2 4 4
 $ gender_id               : Factor w/ 2 levels "Man","Woman": 1 1 2 2 2 1 2 1 1 1 ...
```

```r
#vaccine status ~ political leaning + age + gender
full.model <- glm(covid_vaccination_status ~ age_range +
                    political_leaning + gender_id,
       family = binomial(link = "logit"), data = modelData)
summary(full.model)
```

```
Call:
glm(formula = covid_vaccination_status ~ age_range + political_leaning +
    gender_id, family = binomial(link = "logit"), data = modelData)

Coefficients:
                                    Estimate Std. Error z value Pr(>|z|)
(Intercept)                           2.3218     0.2964   7.834 4.72e-15 ***
age_range21-25                        0.3395     0.1767   1.921  0.05470 .
age_range26-35                        0.3681     0.1802   2.042  0.04115 *
age_range36-45                        0.5815     0.1948   2.986  0.00283 **
age_range46-55                        0.8160     0.1920   4.251 2.13e-05 ***
age_rangeolder than 55                1.4831     0.2260   6.561 5.34e-11 ***
political_leaningLiberal              1.0559     0.3335   3.166  0.00154 **
political_leaningMiddle-of-the-road  -0.7704     0.2856  -2.697  0.00699 **
political_leaningConservative        -2.2812     0.2818  -8.097 5.65e-16 ***
political_leaningFar right           -2.8086     0.4083  -6.879 6.04e-12 ***
gender_idWoman                       -0.1027     0.1190  -0.863  0.38811
---
Signif. codes:  0 '***' 0.001 '**' 0.01 '*' 0.05 '.' 0.1 ' ' 1

(Dispersion parameter for binomial family taken to be 1)

    Null deviance: 2501.0  on 3140  degrees of freedom
Residual deviance: 1971.1  on 3130  degrees of freedom
AIC: 1993.1

Number of Fisher Scoring iterations: 6
```

Since *gender* is not significant adjusted for *political leaning* and *age*, we remove it from the model and obtain a reduced model.

```r
#vaccine status ~ political leaning + age
red.model <- glm(covid_vaccination_status ~ age_range + political_leaning,
       family = binomial(link = "logit"), data = modelData)
summary(red.model)
```

```

Call:
glm(formula = covid_vaccination_status ~ age_range + political_leaning,
    family = binomial(link = "logit"), data = modelData)

Coefficients:
                                   Estimate Std. Error z value Pr(>|z|)
(Intercept)                          2.2638     0.2883   7.852 4.09e-15 ***
age_range21-25                       0.3531     0.1760   2.006  0.04486 *
age_range26-35                       0.3708     0.1802   2.058  0.03957 *
age_range36-45                       0.5813     0.1947   2.985  0.00284 **
age_range46-55                       0.8039     0.1913   4.202 2.65e-05 ***
age_rangeolder than 55               1.4695     0.2254   6.520 7.01e-11 ***
political_leaningLiberal             1.0470     0.3333   3.142  0.00168 **
political_leaningMiddle-of-the-road -0.7747     0.2855  -2.713  0.00667 **
political_leaningConservative       -2.2770     0.2816  -8.085 6.23e-16 ***
political_leaningFar right          -2.7917     0.4075  -6.851 7.34e-12 ***
---
Signif. codes:  0 '***' 0.001 '**' 0.01 '*' 0.05 '.' 0.1 ' ' 1

(Dispersion parameter for binomial family taken to be 1)

    Null deviance: 2501.0  on 3140  degrees of freedom
Residual deviance: 1971.9  on 3131  degrees of freedom
AIC: 1991.9

Number of Fisher Scoring iterations: 6
```

```r
#odds ratio and CIs
exp(cbind(Odds_Ratio = coef(red.model), confint(red.model, level = 0.95)))
```

```
                                    Odds_Ratio      2.5 %     97.5 %
```

```
(Intercept)                         9.61936715 5.64192084 17.5927645
age_range21-25                      1.42348412 1.00913096  2.0131873
age_range26-35                      1.44891707 1.01905158  2.0664503
age_range36-45                      1.78831866 1.22516006  2.6311997
age_range46-55                      2.23432245 1.54111962  3.2658489
age_rangeolder than 55              4.34710786 2.82458581  6.8487433
political_leaningLiberal            2.84923280 1.44991180  5.4096131
political_leaningMiddle-of-the-road 0.46083903 0.25341361  0.7821241
political_leaningConservative       0.10259194 0.05677016  0.1725185
political_leaningFar right          0.06131853 0.02694192  0.1340584
```

Based on the results we have sufficient statistical evidence at 5% level to conclude that odds of being vaccinated is different between ones identified as far-left, and any other political leaning adjusted for age. Adjusted for age the odds of getting vaccines are about three times higher for liberals than the ones identify as far-left (1.45, 5.41). Adjusted for age, people leaning towards far-right political opinion are 94% less likely to be vaccinated than the one identify as far-left (0.027, 0.134).
