# Supplementary material for: Age and political leaning predict COVID-19 vaccination status at a large, multi-campus, public university in Pennsylvania: A cross-sectional survey
Source: PLoS One. 2023 Sep 20;18(9):e0291974. doi: 10.1371/journal.pone.0291974 (PMC10511139; doi:10.1371/journal.pone.0291974)
Supplement: Attachment — Submitted filename: Response to Reviewers (1).docx [file pone.0291974.s003.docx]

Dear Binod Acharya and PLOS One,

Thank you for the thoughtful comments regarding the manuscript “Age and political leaning predict COVID-19 vaccination status at a large, multi-campus, public university in Pennsylvania: a cross-sectional survey” [PONE-D-23-10814].

After addressing the reviewers’ comments, we believe the manuscript more clearly communicates our findings and their implications. Below, you will find how each reviewer’s comments were addressed.

Of note, during the writing of the manuscript, our faculty mentor, Bradley A. Long, was on full-time leave due to a serious family illness. He remains on part time leave, caring for his family member. A few changes to the manuscript not originally asked for were in part due to Mr. Long's post-review contributions.

| **Comment** | **How Comment was Addressed** |
| --- | --- |
| Please ensure that your manuscript meets PLOS ONE's style requirements, including those for file naming. | Done. |
| We note that the grant information you provided in the ‘Funding Information’ and ‘Financial Disclosure’ sections do not match. | Updated Funding Statement added to the cover letter: “The study was funded by an internal grant from the Pennsylvania State University Libraries Faculty Organization. There is no grant number associated with this award, only the year that the grant was issued, 2021.” |
| When you resubmit, please ensure that you provide the correct grant numbers for the awards you received for your study in the ‘Funding Information’ section. | There are no grant numbers associated with any funding we received. |
| Thank you for stating the following in the Acknowledgments Section of your manuscript:  “. RM and LP received funding from The Harrell Health Sciences Library at the Penn State College of Medicine to receive compensation as research assistants. The funders had no role in study design, data collection and analysis, decision to publish, or preparation of the manuscript.”  We note that you have provided additional information within the Acknowledgements Section that is not currently declared in your Funding Statement. Please note that funding information should not appear in the Acknowledgments section or other areas of your manuscript. We will only publish funding information present in the Funding Statement section of the online submission form. | The Funding Statement has been updated and included in the cover letter (see above). All funding information has been removed from the manuscript. |
| Please remove any funding-related text from the manuscript and let us know how you would like to update your Funding Statement. Currently, your Funding Statement reads as follows:  “RM and LP received funding from The Harrell Health Sciences Library at the Penn State College of Medicine to receive compensation as research assistants. The funders had no role in study design, data collection and analysis, decision to publish, or preparation of the manuscript.”  Please include your amended statements within your cover letter; we will change the online submission form on your behalf. | We removed funding-related text from the manuscript and added a Funding Statement to the cover letter. |
| Please note that we encourage authors to report confidence intervals (not just the p-values) to convey the uncertainty of the estimates. | Confidence intervals are now included. |
| Also, please be reminded that the current presentation of results (in Table 5) is not articulated well enough (both in terms of substance and format requirement of the journal). Importantly, readers might want to see the results of the association between hesitancy and demographic factors of interest when simultaneously controlling for all of them (i.e. age and political leaning and maybe more covariates about which you have collected the data). Also, note that some of the numbers presented do raise eyebrows. For example, the ORs comparing far-light versus far-left is as small as 0.06 (the back-of-the-envelope calculation of unadjusted ORs gives a very different number). Please also attach the associated R code during the revised submission. | The substance and format of Table 5 has been updated.  The R-code is included in the submission. |
| In the abstract: there appears to be a typo on OR (4.26 vs 4.30). Again, I note that the associated P with (big) OR >4 is 0.04. Please report the width of CI as well. | Typos have been addressed and Cis are now included. |
| Moreover, please be reminded that we do not do copy editing and it’s your responsibility to address any and all typos. A few examples: “Conversative” for conservative; “R™” with unnecessary trademark, and “COVID” without 19. | Conversative replaced with conservative.  R trademark removed.  COVID replaced with COVID-19. |
| Specific comments on the text. Please note that this sentence below gives an impression of as if Table 1,3,4 present associations. They don’t. Also redundant Table 1. And you didn't "made" associations.  “Associations between selected factors such as gender (Table 1), age (Table 1), political orientation (Table 3) and vaccine hesitancy (Table 4) were made with Chi-square tests and generalized linear regression models on R™ version 4.2.1 (2022-06-23).” | Sentence changed to:  “Chi-square tests and generalized linear regression models on R version 4.2.1 (2022-06-23) were used to evaluate associations between vaccine hesitancy (Table 4) and selected survey factors (gender, age, and political orientation).” |
| Also, given the specific study sample is the PSU, what exactly do authors have in mind when you say “public health efforts such as vaccine campaigns and policy decisions” in your conclusion. | We agree we could clarify the conclusion. Our intention was to say our single-institution study may help influence decisions made on campus regarding public health efforts.  Conclusion changed to:  “Age and political leaning are key predictors of vaccine uptake among members of the PSU community, knowledge of which may inform campus leadership’s public health efforts such as vaccine campaigns and policy decisions.” |
| First, the manuscript does not provide a literature review on the association between vaccine incentive programs and vaccine uptake in the United States. The authors should include a comprehensive review of relevant studies in the introduction and discussion sections. Please refer to the following two articles.  [https://jamanetwork.com/journals/jamanetworkopen/article-abstract/2786986](https://urldefense.com/v3/__https:/jamanetwork.com/journals/jamanetworkopen/article-abstract/2786986__;!!Ls64Rlj6!26a5tfthTnV2OYc04Ss-qqb5es5CjQTMaGTBJfU6VNR61sDr8-m9loYCVOzwEHCpCCG8CWpdDNtgYe-oNZVkom_ifg$)  [https://academic.oup.com/aje/article/192/4/510/6987267](https://urldefense.com/v3/__https:/academic.oup.com/aje/article/192/4/510/6987267__;!!Ls64Rlj6!26a5tfthTnV2OYc04Ss-qqb5es5CjQTMaGTBJfU6VNR61sDr8-m9loYCVOzwEHCpCCG8CWpdDNtgYe-oNZViltFETw$) | In the third paragraph of the discussion, vaccine incentives and both listed articles are now mentioned:  “Future projects would involve piloting interventions, such as vaccine incentive programs, that target the groups with lower vaccination rates and assessing if vaccine uptake meaningfully increased within those groups. Given the compelling evidence supporting vaccination to reduce mortality from COVID-19, and the emergence of novel COVID-19 variants, it is in the best interest of PSU to strategize ways to increase vaccine uptake (17). Although not trialed at PSU, vaccine lotteries have shown efficacy in increasing uptake, although with mixed success depending on geographic location (i.e., state) (Acharya and Dhakal, 2021). Booster dose uptake is more likely to be influenced by vaccine lottery incentives than initial doses (McLaren, 2023). Both findings highlight the importance of utilizing heterogenous approaches to increasing vaccine uptake, considering both geographic factors as well as the vaccine dose type, when formulating incentives.” |
| Second, the authors did not report the response rate of the survey, which is important to assess the representativeness of the sample. | The response rate was added:  “4,231 survey responses were received. 32,792 emails were randomly sent to those with a PSU email address, making the response rate 12.90%.” |
| Third, the study design is cross-sectional, which makes it difficult to establish a causal relationship between the factors investigated and vaccine hesitancy. In addition, the authors did not provide a detailed description of the recruitment methods, which makes it difficult to assess the potential for selection bias. The authors should acknowledge these limitations and discuss how they may have affected the results and the generalizability of the findings. | The recruitment methods have been clarified:  “Participants were recruited by randomly emailing 32,792 individuals with a PSU email address. Additionally, digital and print marketing campaigns took place with campus websites, bulletins, and newspapers.”  The following points were added to the discussion:  “The cross-sectional design of the study hinders the ability to establish a causal relationship between vaccine hesitancy and the selected demographic factors.”  “Other potential confounders such as education level, race/ethnicity and health status were also not controlled for when assessing vaccine hesitancy.” |
| Fourth, the authors did not control for other potential confounders that may be associated with vaccine hesitancy, such as education level, race/ethnicity, and health status. Was there any effort to measure or control for the influence of social media and other external sources of information on vaccine hesitancy or to assess participants' trust in data from various sources? | The following point was added to the discussion:  “Other potential confounders such as education level, race/ethnicity and health status were also not controlled for when assessing vaccine hesitancy.”  The reviewer brings up an excellent point about the influence of external sources of information on vaccine hesitancy. The survey did in fact inquire about social media usage and how participants obtain health-related news information. Participants were explicitly asked if they trusted messages from government experts/agencies. However, the authors feel it is appropriate to report these data in future studies rather than the current one. |
| Fifth, the manuscript lacks a clear discussion of the practical implications of the findings for public health policy and practice. The authors should provide a detailed discussion of the implications of their findings for vaccine campaigns and policy decisions, and they should also discuss the potential for future research in this area. | The following points were added:  “Further analysis revealed that those aged 56 years or older and those of liberal political leanings were more likely to report COVID-19 vaccination. Knowledge of this has the potential to influence leadership’s approach to ensuring public health on campus. For example, communications primarily targeting the 56+ year old and/or liberal-leaning political groups may not be as fruitful as those targeting groups with lower vaccine uptake. Given the compelling evidence supporting vaccination to reduce mortality from COVID-19, it is in the best interest of PSU to strategize ways to increase vaccine uptake in such groups (17). Future projects would involve piloting interventions that target the groups with lower vaccination rates and assessing if vaccine uptake meaningfully changed within those groups. Especially with the emergence of novel COVID-19 variants and approval of bivalent vaccines, targeted communication and further research are needed.”  “Specifically, the authors aim to explore how trust in government agencies, trust in elected officials, and methods of obtaining health-related news information (social media versus print newspapers, for example) influence individual decisions to receive a vaccine against COVID-19.” |
| The article lacks clarity regarding the methodology employed to identify the political preferences of the respondents. The authors should provide a detailed explanation of the approach utilized to collect and categorize political leaning information.  It would be beneficial to include information on how the potential bias or subjectivity in assessing political preference was minimized or accounted for in the analysis.  The authors should consider providing additional information about the political spectrum utilized in the study to help readers understand the categories and their definitions. | Further commentary about methodology was included in the methods section:  “Political orientation was measured by modifying the three-point Gallup item. Rather than asking if participants are Republican, Democrat, or Independent, they were asked to identify with one of the following: Far left, Liberal, Middle-of-the-road, Conservative, Far right, or Prefer not to answer. The authors believe avoiding specific political parties and expanding the number of responses captures more granularity than the three-point Gallup item.”  Additionally, the limitations of the political scale that was used is also mentioned in the discussion:  “Participants may have had difficulty selecting survey responses if no clear definitions were provided, which was the case for the political leaning question, for example.” |
| The article would benefit from a more explicit discussion of the specific concerns related to the Johnson & Johnson vaccine, such as the reported cases of blood clotting and the subsequent FDA advisory. Providing a clear overview of these concerns and their potential impact on vaccine hesitancy would enhance the readers' understanding. | The reviewer addresses a great point that participants may have had varying degrees of safety concerns based on the specific type of vaccine they received. Unfortunately, the survey did not inquire about this. There are other pieces of context that make any analysis about vaccine brand difficult; for the most part, PSU mostly provided Moderna vaccines. Since employees are penalized for not being vaccinated, the authors believe there are too many confounders to use vaccine brand as a hesitancy factor.  A study by Merkley supports the reviewer’s point that there has been hesitancy towards particular vaccines (Merkely).  As our survey was in development, various vaccines were also being developed and approved at various times (CDC).  Additionally, availability of particular vaccines at the time of the survey varied by community and healthcare provider within Pennsylvania, limiting individual choice at some campuses (Simmons-Ritchie).  Therefore, this aspect of vaccine hesitancy was not considered for inclusion within the survey.    Merkley E, Loewen PJ. The correlates and dynamics of COVID-19 vaccine-specific hesitancy. Vaccine. 2022;40(13):2020–7. Available from: <https://www.sciencedirect.com/science/article/pii/S0264410X22001682>    CDC museum COVID-19 timeline [Internet]. Centers for Disease Control and Prevention. 2023 [cited 2023 Jul 26]. Available from: <https://www.cdc.gov/museum/timeline/covid19.html>    Simmons-Ritchie D. Where to get the COVID-19 vaccine in Pennsylvania and who can get it (discontinued) [Internet]. Spotlight PA. 2021 [cited 2023 Jul 26]. Available from: <https://www.spotlightpa.org/news/2021/01/pa-covid-vaccine-locations-availability-where-to-get-who-can-get-latest-updates/> |

Thank you for your time and consideration. Your comments strengthened our manuscript.

Sincerely,

**Ryan Murphy
Lauren Pomerantz
Prabhani Kuruppumullage Don, PhD
Jun Sung Kim
Bradley A. Long, MSLS**
